# Enhanced chondrogenic potential in GelMA-based 3D cartilage model via Wnt3a surface immobilization

**DOI:** 10.1038/s41598-024-65970-w

**Published:** 2024-07-01

**Authors:** Angela Imere, Nicola C. Foster, Hadi Hajiali, Kerime Ebrar Okur, Abigail L. Wright, Ines A. Barroso, Alicia J. El Haj

**Affiliations:** Healthcare Technologies Institute, Institute of Translational Medicine, National Institute for Health and Care Research (NIHR) Birmingham Biomedical Research Centre, School of Chemical Engineering, University of Birmingham, Birmingham, B15 2TT UK

**Keywords:** Stem cells, Mesenchymal stem cells, Regeneration, Stem-cell differentiation, Biomedical engineering, Drug development, Stem-cell research

## Abstract

Cartilage tissue engineering aims to develop functional substitutes for treating cartilage defects and osteoarthritis. Traditional two-dimensional (2D) cell culture systems lack the complexity of native cartilage, leading to the development of 3D regenerative cartilage models. In this study, we developed a 3D model using Gelatin Methacryloyl (GelMA)-based hydrogels seeded with Y201 cells, a bone marrow mesenchymal stem cell line. The model investigated chondrogenic differentiation potential in response to Wnt3a stimulation within the GelMA scaffold and validated using known chondrogenic agonists. Y201 cells demonstrated suitability for the model, with increased proteoglycan content and upregulated chondrogenic marker expression under chondrogenic conditions. Wnt3a enhanced cell proliferation, indicating activation of the Wnt/β-catenin pathway, which plays a role in cartilage development. GelMA hydrogels provided an optimal scaffold, supporting cell viability and proliferation. The 3D model exhibited consistent responses to chondrogenic agonists, with TGF-β3 enhancing cartilage-specific extracellular matrix (ECM) production and chondrogenic differentiation. The combination of Wnt3a and TGF-β3 showed synergistic effects, promoting chondrogenic differentiation and ECM production. This study presents a 3D regenerative cartilage model with potential for investigating cartilage biology, disease mechanisms, and drug screening. The model provides insights into complex cartilage regeneration mechanisms and offers a platform for developing therapeutic approaches for cartilage repair and osteoarthritis treatment.

## Introduction

Cartilage tissue engineering is a promising field that aims to develop functional cartilage substitutes for the treatment of cartilage defects and osteoarthritis. Traditional two-dimensional (2D) cell culture systems have limitations in recapitulating the complex three-dimensional (3D) architecture and cellular microenvironment of native cartilage^1^. Within cartilage, a diverse array of cells exists, intricately arranged to form a complex tissue structure. Therefore, the development of 3D regenerative cartilage models has gained significant attention in recent years^1,2^. These models offer a more physiologically relevant platform for studying cartilage biology, disease mechanisms, and importantly drug screening applications.

In the field of tissue engineering, hydrogels have emerged as promising scaffolds due to their biocompatibility, tunable mechanical properties, and ability to encapsulate cells and bioactive molecules^3–6^. Gelatin methacryloyl (GelMA) is a commonly used hydrogel that exhibits excellent biocompatibility and can be easily functionalized to incorporate bioactive cues^4,7,8^. GelMA-based hydrogels have shown great potential for cartilage tissue engineering applications such as drug screening^9–11^. They provide a compatible and scaleable microenvironment for enhanced chondrogenic cell viability and functionality^12^. These characteristics make GelMA an attractive choice for scaffold fabrication in 3D regenerative cartilage models.

Furthermore, the Wnt signaling pathway plays a critical role in cartilage development, homeostasis, and regeneration^13^. Activation of Wnt signaling, particularly the Wnt/β-catenin pathway, has been shown to play a dual role in modulation of chondrogenic differentiation and the inhibition of chondrocyte hypertrophy^13^. Wnt3a, a Wnt signaling agonist, has been widely investigated for its potential in cartilage tissue engineering^14,15^. It has also been shown to exert significant influence on asymmetric cell division and cell migration, playing pivotal roles in the intricate orchestration of developmental and physiological events^16,17^. It is noteworthy that soluble Wnt3a, when added globally to cells, lacks the spatial cues, which have been found to play a role in asymmetric division during differentiation^16,17^. Our studies have demonstrated that the immobilization of Wnt3a promotes asymmetric division of embryonic stem cells and skeletal stem cells, providing crucial directional cues for engineering 3D tissues^18,19^. Wnt3a serves as a potent activator of the Wnt-β-catenin pathway, as evidenced by previous studies^14,20^. While an excessive activation of this pathway in healthy cartilage has been implicated in cartilage breakdown^21,22^, its role becomes crucially different following cartilage injury. In such instances, when tissue remodelling and cell expansion are necessary for repair, an appropriately timed upregulation of Wnt3a has been associated with enhanced tissue repair and regeneration, as shown in the research by Cheverud et al*.*^23^. These findings highlight the context-dependent nature of Wnt3a signalling in cartilage biology and underscore its potential as a key regulator in cartilage tissue engineering and regenerative medicine approaches.

Moreover, in cell-based cartilage regenerative models, primary chondrocytes have traditionally been employed. However, the scarcity of donor sites and the relatively low yield of isolated cells from autologous tissue (representing only 1–5% of the total tissue volume) pose significant challenges^24^. To address these limitations, there has been a growing interest in investigating the differentiation of immortalized stem cells into chondrocytes as a promising alternative^25^. This approach offers several advantages, including cost-effectiveness, improved reproducibility, enhanced proliferative capacity, and faster production timelines, making it an attractive option for the development of cartilage tissue engineering models for high throughput screening applications^25^. Y201 cells, a type of bone marrow mesenchymal stem cells (MSCs)^26^, possess the ability to undergo chondrogenic differentiation and has been demonstrated for cartilage regeneration models^27,28^.

**Figure 1:**
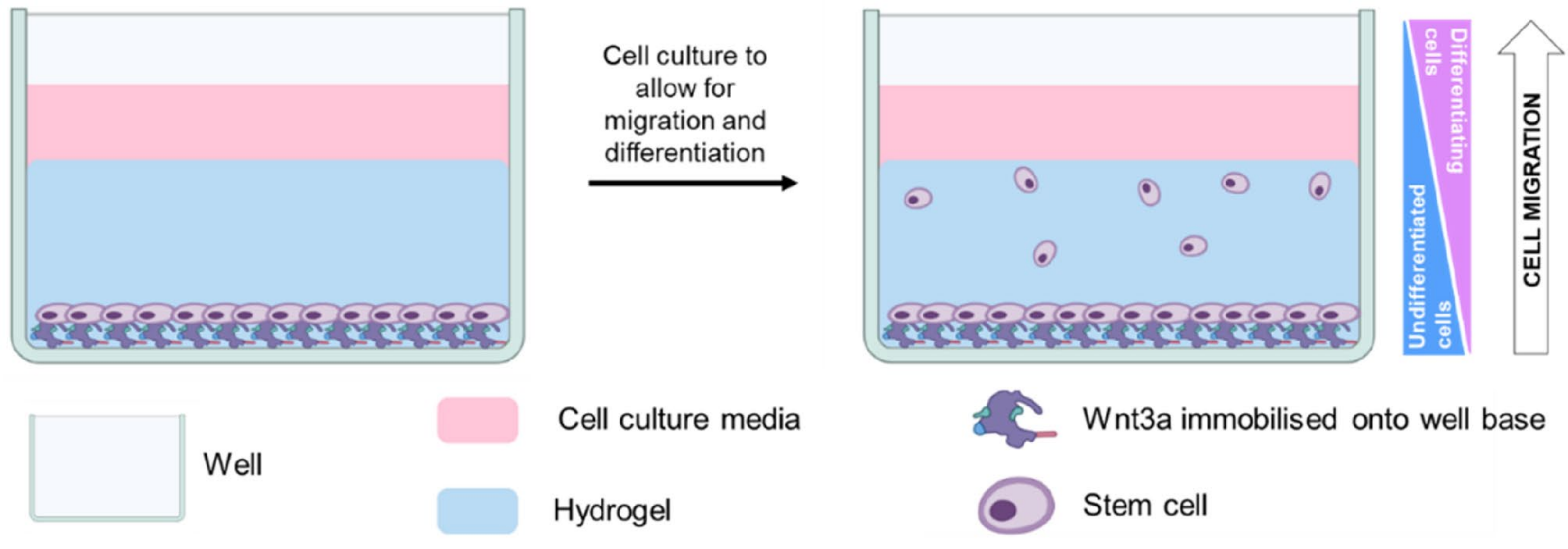
Schematics of 3D regenerative cartilage model. Stem cells are seeded in monolayer into a 96-well plate, which was previously chemically modified to allow immobilization of Wnt3a onto the base of the well, covered with a hydrogel. Then, the sample is covered in medium (either basic medium or supplemented with the chondrogenic molecule of interest) and cultured to allow for cell migration towards the top of the gel and differentiation. After the culture period, cells at the bottom of the well maintain their stemness, while as they migrate to the top, cells commit to the chondrogenic lineage (cell differentiation level depending on the efficacy of the screened drug).

In existing tissue engineering approaches, there are some limitations such as scalability, reproducibility, and the ability to recapitulate the complex microenvironment of native cartilage tissue. By addressing these limitations, our study aimed to contribute to overcoming current challenges in cartilage tissue engineering. In this study, our aim was to develop a 3D regenerative cartilage model incorporating bioactive cues, such as Wnt3a, using GelMA-based hydrogel seeded with Y201 cells, which can be scaled up for use in drug screening applications (Fig. 1). We aimed to investigate the chondrogenic differentiation potential of Y201 cells in response to Wnt3a stimulation within the 3D hydrogel scaffold. Furthermore, we aimed to assess the production of cartilage-specific extracellular matrix (ECM) and validate the chondrogenic model using known chondrogenic agonists. By integrating 3D regenerative cartilage models including GelMA hydrogels, Wnt3a, and Y201 cells for drug screening, we can advance the field of cartilage tissue engineering, improve our understanding of cartilage regeneration mechanisms, and potentially discover novel therapeutic approaches for cartilage repair and osteoarthritis treatment.

## Materials and methods

### Wnt3a immobilization

Recombinant mouse Wnt3a protein was purchased from R&D Systems and stock solutions, prepared by reconstituting the protein to 10 µg/ml in a solution of 1% CHAPS buffer (Merck), were stored in aliquots at − 80 °C for up to 3 months. Wnt3a working solutions were prepared by diluting the stock solution to 600 ng/ml in Dulbecco’s Phosphate Buffered Solution (DPBS) (Gibco). Wnt3a was immobilized on the surface of 96-well plate (Corning) by adding 40 µl of protein working solution in previously modified wells. Well plate modification was performed by adding a 2% (3-Aminopropyl)triethoxysilane (APTES) (Merck) solution in 90% ethanol (Merck) and incubating for 30 min at room temperature (RT), followed by two washed with 100% ethanol. After 1 h of incubation at RT, Wnt3a solution was removed, followed by a wash with DPBS. For control wells, Wnt3a protein was inactivated after immobilization with a freshly made 20 mM Dithiothreitol (DTT) (Merck) solution in DPBS, which was incubated at 37 °C for 30 min and washed with DPBS. In order to block exposed silane groups, prior to cell culture all wells were covered for 1 h in basic medium, consisting of high glucose Dulbecco’s Modified Eagle Medium (DMEM) supplemented with 10% Fetal Bovine Serum (FBS) (Gibco), 1% L-Glutamine solution (Merck) and 1% penicillin/streptomycin solution (PS) (Merck).

### Y201 cell culture and characterization

#### Cell culture

Y201 bone-marrow stromal cells (BMSCs), kindly donated by Prof. Paul Genever from the University of York, were stably transfected with 7xTCF-GFP/SV40-mCherry as previously described.^26,29^ Cells were routinely passaged every 2–3 days in basic medium when they reached ~ 80% confluency. For all experiments cells were used up to passage 20. For chondrogenic differentiation (also referred to as ‘TGF-β3’ condition), medium consisted of high glucose DMEM supplemented with 1% FBS, 1% L-glutamine, 1% PS, 10% sodium pyruvate solution, 40 μg/ml L-proline, 50 μg/ml L-ascorbic acid-2-phosphate, 10% insulin-transferrin-selenium solution (ITS), 100 nM dexamethasone (all from Merck) and 10 ng/ml recombinant human TGF-β3 (Peprotech). Kartogenin-supplemented medium was prepared using high glucose DMEM supplemented with 5% FBS and 100 nM kartogenin (Selleck Chemicals).

Cells were pelleted and differentiated into chondrocytes using a high-throughput v-bottomed 96-well plate culture system, following previously established protocols^30^. Each well of an autoclave-sterilized v-bottomed 96-well polypropylene microplate (Greiner bio-one) received 200,000 cells at passage 3, which were subsequently centrifuged at 500 × g for 5 min. The resulting cell pellets were cultured in 250 µl of chondrogenic differentiation medium, composed of high glucose DMEM supplemented with 2 mM L-glutamine, 100 U/mL penicillin-0.1 mg/ml streptomycin, 100 μg/mL sodium pyruvate, 40 μg/mL L-proline, 50 μg/mL L-ascorbic acid-2-phosphate, 4.7 μg/mL linoleic acid, 1.5 mg/mL bovine serum albumin (BSA), 1 × insulin-transferrin-selenium, 100 nM dexamethasone (all obtained from Merck), and 10 ng/ml recombinant human TGF-β3 (Peprotech). Throughout the experiment, the culture medium was refreshed three times weekly, and the cell pellets were maintained for a total duration of three weeks.

In order to investigate Y201 cell response to Wnt3a, cells were detached, counted and seeded on 96-well plates at a density of at approximately 5 × 10^4^ cells/cm^2^. Cell were cultured for 4 days in basic medium with/without 50 ng/ml Wnt3a.

#### Histology

Samples of Y201 cell pellets in basic and chondrogenic conditions (n = 2 per group) were fixed in 10% neutral buffered formalin for 2 h and embedded in paraffin. Samples were cut as 5 µm-thick slices and deposited on SuperFrost™ glass slides (ThermoScientific). Sections were de-waxed in xylene for 5 min, followed by re-hydration in descending grades of ethanol to water. Histological slides were stained with Toluidine blue (Merck) for production of proteoglycans and GAGs, and counterstained with Gill’s number 2 Hematoxylin (Merck) according to manufacturer's instructions. Sections were then washed in absolute ethanol thrice and cleared in xylene before mounting with DPX mountant (Merck). Sections were imaged using an EVOS XL Core microscope.

#### Assessment of gene expression

Y201 cell pellets in basic and chondrogenic conditions (n = 3 per group) were snap frozen after 26 days of culture and homogenized using disposable pellet pestles (Merck). RNA was then extracted using TRI Reagent (Merck) and converted into cDNA using high-capacity cDNA Reverse Transcription Kits (Applied Biosystems) (both as per the manufacturer's instructions). Gene expression analysis was performed for ACAN and COL2A1 using SYBR Green-based quantitative real-time polymerase chain reaction (qRT-PCR) with pre-optimized QuantiTect primer assays (Qiagen) and an AriaMx Real-Time PCR System (Agilent Technologies). qRT-PCR data were analyzed using the ΔΔCt method as described previously^31^, with gene expression normalized to the reference gene GAPDH.

### Wnt3a/Gel platform preparation

#### Preparation of hydrogel precursor solutions

For gellan gum hydrogels, a 1% (w/v) solution was prepared by dissolving Phytagel™ (Merck) in dH_2_O. The solution was heated in a microwave for 5 s, followed by manual swirling for 2–3 s and this process was repeated until the powder was completely dissolved (~ 3 min). The precursor solution was UV-sterilized for 90 s and then incubated at 37 °C for at least 30 min to allow for cooling without setting and maintained at 37 °C until needed.

For collagen hydrogels, stock rats tail collagen type I solution (Corning) was diluted to 1 mg/ml using basic medium supplemented with 20% HEPES buffer. The hydrogel solution was maintained in ice to avoid setting of the gel until needed.

For Poly(ethylene glycol) (PEG) hydrogels, a 5% (w/v) solution was prepared by dissolving Poly(ethylene glycol) diacrylate pellets (ThermoFisher) in DPBS. Then, Irgacure® 1173 (BASF) photoinitiator was added (1 µl in 1 ml of PEG solution) and the precursor solution was left in the dark until needed.

For GelMA hydrogels, polymer synthesis was performed as previously described.^32^ Briefly, a 10% (w/v) solution was prepared by dissolving gelatine type A from porcine skin (300 bloom strength, Merck) in DPBS at 60 °C for 30 min. Methacrylic anhydride (MA) (Merck) was added to the gelatine solution to a final concentration of 6% (v/v) and left to react for 2 h at 50 °C under vigorous stirring. After the reaction period, the solution was transferred to 50 mL tubes and the unreacted MA was partially removed by centrifugation at 3500 rpm for 5 min at RT. The solution was then dialyzed against distilled water for 7 days at 37 °C using 12–14 kDa cut-off dialysis tubes (Thermo Scientific). After dialysis, the GelMA solution was diluted to 2% (w/v) and the pH adjusted to 7.4 using 1 mM sodium hydroxide solution (Merck). Lastly, GelMA solutions were lyophilized for 2 days to generate a white porous foam, which was stored at − 80 °C until further use. After GelMa synthesis, a 4% (w/v) hydrogel precursor solution was prepared by dissolving the synthesized polymer in DPBS at 60 °C for 30 min and UV-sterilized for 5 min. Then, 40 mM phosphated riboflavin (Merck) and 546 mM sodium persulphate (Merck) solution were prepared in dH_2_O and filter-sterilized. The two photoinitiators were added into the GelMA solution at a final concentration of 2 mM and 10 mM for riboflavin and sodium persulfate, respectively, and maintained in the dark until needed.

#### Set-up of the 3D model

Y201 cells were detached, resuspended in serum-free medium and seeded onto Wnt3a-immobilized 96-well plates at a density of 3 × 10^4^ cells per well. Cells were left to adhere for at least 1 h before the medium was removed and the cells were covered with 50 µl per well of the appropriate hydrogel solution. To allow for gelation, gellan gum and collagen precursor solutions were incubated for 10 min at 37 °C, while PEG samples were irradiated with broad spectrum UV light for 5 min at a distance of 1.7 cm. GelMA precursor solution was set using a 100 mW/cm^2^ Knightsbridge FLF Floodlight visible light lamp (RS Components) for 5 min at a distance of 5.5 cm. After the hydrogels were set, 200 µl of appropriate medium was added on top of the gels and samples were incubated at 37 °C and 5% CO_2_ for 1 h, before the medium was replaced. Basic, chondrogenic or kartogenin-supplemented medium (described above in Section "Cell culture") was used for samples in basic, ‘TGF-β3’ and ‘kartogenin’ conditions, respectively. After 72 h of culture, kartogenin-supplemented medium was replaced with high glucose DMEM supplemented with 5% FBS, 1% L-Glutamine, 1% PS and 1% ITS. For all conditions, during the culture period medium was replaced every other day.

### Optical coherence tomography (OCT)

4% (w/v) GelMA hydrogels were prepared in 96-well plate as described in Section "Preparation of hydrogel precursor solutions". Prepared hydrogels were covered with culture medium to simulate the experimental conditions and underwent OCT imaging on days 0, 3, and 7. A 20 µl medium was added while doing the imaging in order to prevent dehydration and reflection. Following the imaging procedure, 200 µl of the appropriate culture medium was added onto the hydrogels, and the samples were subsequently incubated at 37 °C. Mechanical property analysis was carried out utilizing debiased ambient vibration Optical Coherence Elastography (OCE). This technique, designed for mechanical contrast evaluation, was conducted using an OCT imaging system, with no supplementary hardware modifications. This approach allows for the non-contact, sterile assessment of mechanical properties, relying on using ambient vibrations. The OCT system employed in this study featured a lateral scanning distance of approximately 3.7 mm. Mechanical property assessment encompasses the generation of mechanical contrast maps and quantitative analysis through the calculation of Young's modulus (E). E is derived using a calibration curve established through nanoindentation and OCT imaging. The correlation between E and the estimated wavelength (λ) follows the equation E ∝ λ^2^, which justifies the utilization of the mean wavelength squared, λ^2^, in E calculation^33^.

### Biochemical assays

Y201 cells cultured in monolayer (n = 4 per group) were washed with DPBS, detached and centrifuged to form a pellet, while the 3D cell constructs (n = 4 per group) were only washed with DPBS. 250 µL of Proteinase K (Invitrogen) (1 mg/mL dissolved in 100 mM ammonium acetate, pH 7.0) was added per 100 mg wet weight of sample. Samples were vortexed, then incubated at 60 °C with an additional vortex every 30 min until complete dissociation had occurred (~ 2 h). Proteinase K was inactivated by heating the samples to 100 °C for 5 min in a heat block. DNA content was quantified using the PicoGreen dye assay (Biosciences) following the kit instructions with a calf thymus DNA standard. Sulfated glycosaminoglycan (sGAG) content within the sample was quantified using the dimethylmethylene blue (DMMB) dye-binding assay with a chondroitin sulfate standard. The sGAG content secreted into media was analyzed using Glycosaminoglycan Assay Blyscan kit (Biocolor) following the kit instruction, also with a chondroitin sulfate standard. Cell proliferation in monolayers was quantified using the EdU (Abcam) and Ki67 (Abcam) assays following the kit instructions.

### Immunofluorescence staining

Y201 cells were either cultured in monolayers at a seeding density of approximately 5 × 10^4^ cells/cm^2^ or 5 × 10^4^ cells/ml in a 96-well plate for 7 days or in 3D hydrogels for 7–14 days, as described above. Samples were fixed in 10% neutral buffered formalin for 15 min (cell monolayers) or 2 h (cells in hydrogels) and simultaneously permeabilized and blocked with 2% bovine serum albumin (BSA)/0.1% Triton-X in PBS for 2 h. Cells were stained for STRO-1 (Abcam), SOX9 (Abcam), NCAM (R&D Systems) and COL2A1 (Merck). All primary antibodies were diluted in 1% BSA/0.1% Triton-X at a working concentration of 10 µg/ml and incubated overnight at 4 °C. After washing with PBS, cells were labelled with donkey anti-rabbit AlexaFluor488 (ThermoFisher), donkey anti-goat AlexaFluor568 (ThermoFisher) and/or donkey anti-mouse AlexaFluor 647 (ThermoFisher) diluted at a working concentration of 2 µg/ml in 1% BSA/0.1% Triton-X and incubated for 2 h at RT. Cells were washed with PBS thrice, counterstained with DAPI (Merck) for 15 min and stored in PBS. Fluorescence microscopy was performed on an EVOS M5000 microscope for cell monolayers, while a confocal Olympus IX81 microscopewas used to image 3D hydrogels. Based on the number of migrated cells, the migration results were categorized. If there were < 10 cells, this was categorized as poor migration as that was a relatively low proportion of the total in the bottom. If more than 10 cells were observed, this was categorized as average migration, while migration that exceeded 400 µm in the 500 µm analysis was considered as good migration.

### Statistical analysis

Statistical analysis was performed using GraphPadPrism software package (Dotmatics). Unpaired t-test was used to compare between two groups. Statistical significance was considered if *p* < 0.05. Data are presented as mean ± standard deviation (SD).

## Results

### Y201 cell line for model development

**Figure 2:**
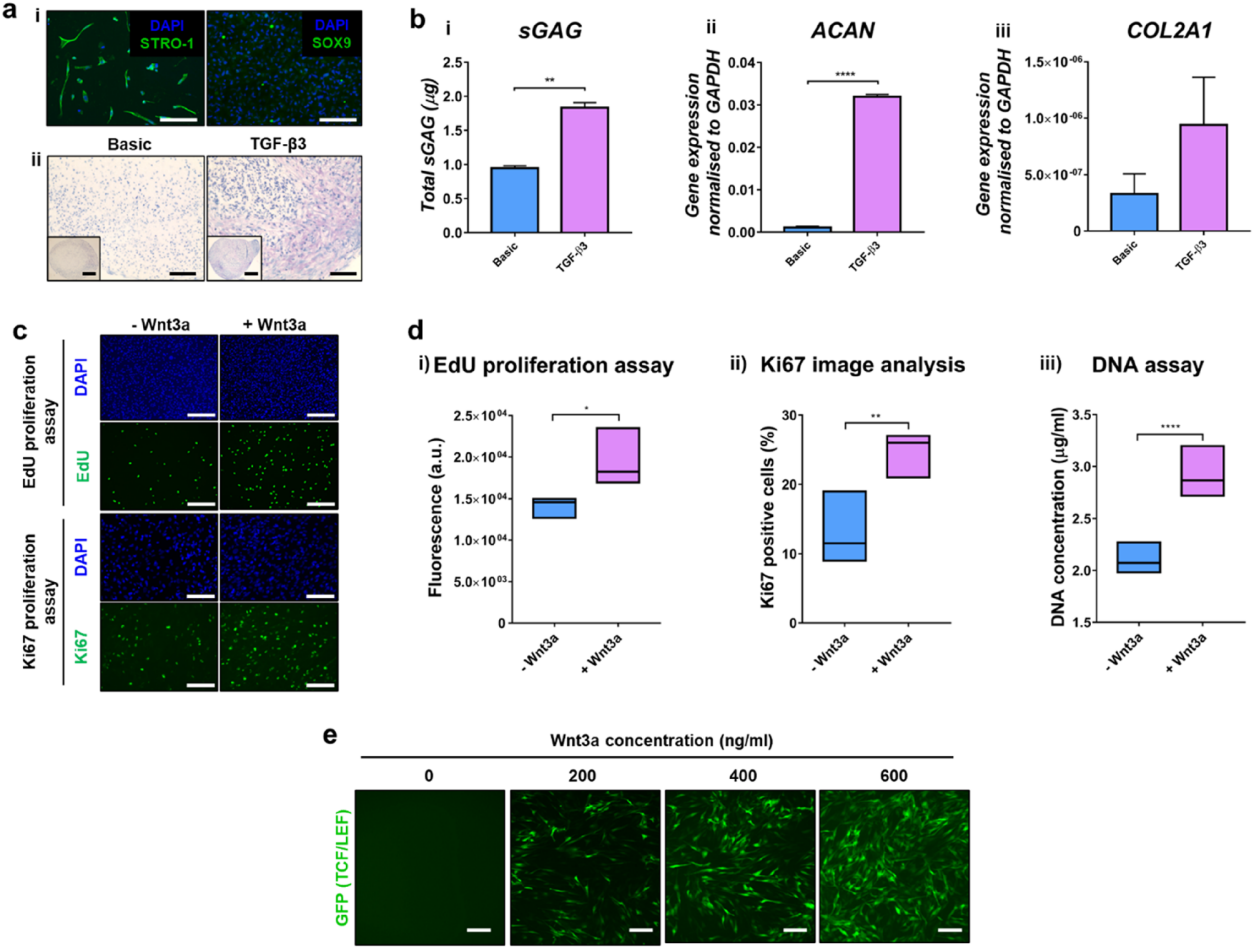
Characterization of Y201 GFP TCF/LEF reporter BMSCs, showing their suitability as a cell source for the development of a 3D regenerative cartilage model for drug screening applications. (**a**) (i) Representative immunofluorescence images of undifferentiated Y201 cells cultured in monolayer for 7 days, showing cell nuclei (blue) and high expression of STRO-1 (left image, green) and low levels of SOX9 (right image, green), consistent with the undifferentiated state of Y201 cells. (ii) Y201 cell pellet in basic (left) and chondrogenic conditions (right) after 26 days of culture stained for proteoglycans, showing increased production of proteoglycans in presence of TGF-β3 compared to the control. Scale bars = 150 µm. (**b**) (i) sGAG assay indicates significant increase in sGAG production in chondrogenic medium compared to basic control. (ii) Gene expression of ACAN and (iii) COL2A1 relative to GAPDH indicates a cartilage-like genetic profile of the cells under chondrogenic conditions (**p < 0.01; ****p < 0.0001). (**c**) Representative immunofluorescence images of EdU and Ki67 proliferation assay, showing improved proliferation of Y201 monolayers exposed to soluble Wnt3 (+ Wnt3a) compared to untreated control (-Wnt3a) (nuclei = blue, Edu/Ki67 = green). Scale bars = 150 µm. (**d**) Quantification of (i) Edu, (ii) Ki67 and (iii) Pico Green assays, confirming enhanced proliferation of Y201s upon treatment with soluble Wnt3a (*p < 0.05; **p < 0.01; ****p < 0.0001). (**e**) Representative immunofluorescence images of Y201 BMSCs confirming Wnt3a functionality by activation of the TCF/LEF reporter (GFP TCF/LEF = green). Cell response to Wnt3a is dose dependent. Scale bars = 150 µm.

Y201 BMSCs with GFP TCF/LEF reporter were characterized to ensure their suitability as a reproducible cell source in our chondrogenic model. Stemness is confirmed in undifferentiated cells cultured in monolayer, since expression of the mesenchymal stem cell marker STRO-1 is observed, while low levels of the chondrogenic marker SOX9 is detected (Fig. 2ai). Y201 MSCs were cultured for 3 weeks in a cell pellet under both basic and chondrogenic conditions (i.e. with TGF-ꞵ3). In presence of TGF-ꞵ3, cells produce a cartilaginous matrix, having high amounts of proteoglycans (Fig. 2aii) compared to the basic control and this outcome is confirmed by the sGAG assay (Fig. 2bi). Key chondrogenic markers ACAN and COL2A1 are overexpressed in chondrogenic conditions (Fig. 2bii,iii). These outcomes highlight the ability of the selected cell line to switch from a mesenchymal stem cell behavior to a differentiated chondrogenic lineage, depending on the environmental conditions.

Wnt3a activity on Y201 BMSCs was also investigated. Fig. 2c demonstrates that supplementation of Wnt3a in the medium significantly improves cell proliferation compared to the untreated control, as also confirmed by quantification of Edu (Fig. 2di), Ki67 (Fig. 2dii) and DNA (Fig. 2diii) assays. Moreover, transfection of Y201 cells with GFP TCF/LEF reporter allows assessment of the activation of the Wnt/β-catenin signalling pathway. Results show that TCF/LEF reporter is activated by Wnt3a and cell response is dose dependent, with stronger reporter activity at higher Wnt3a doses (Fig. 2e).

### Selection of GelMA as hydrogel for 3D cartilage model

**Figure 3:**
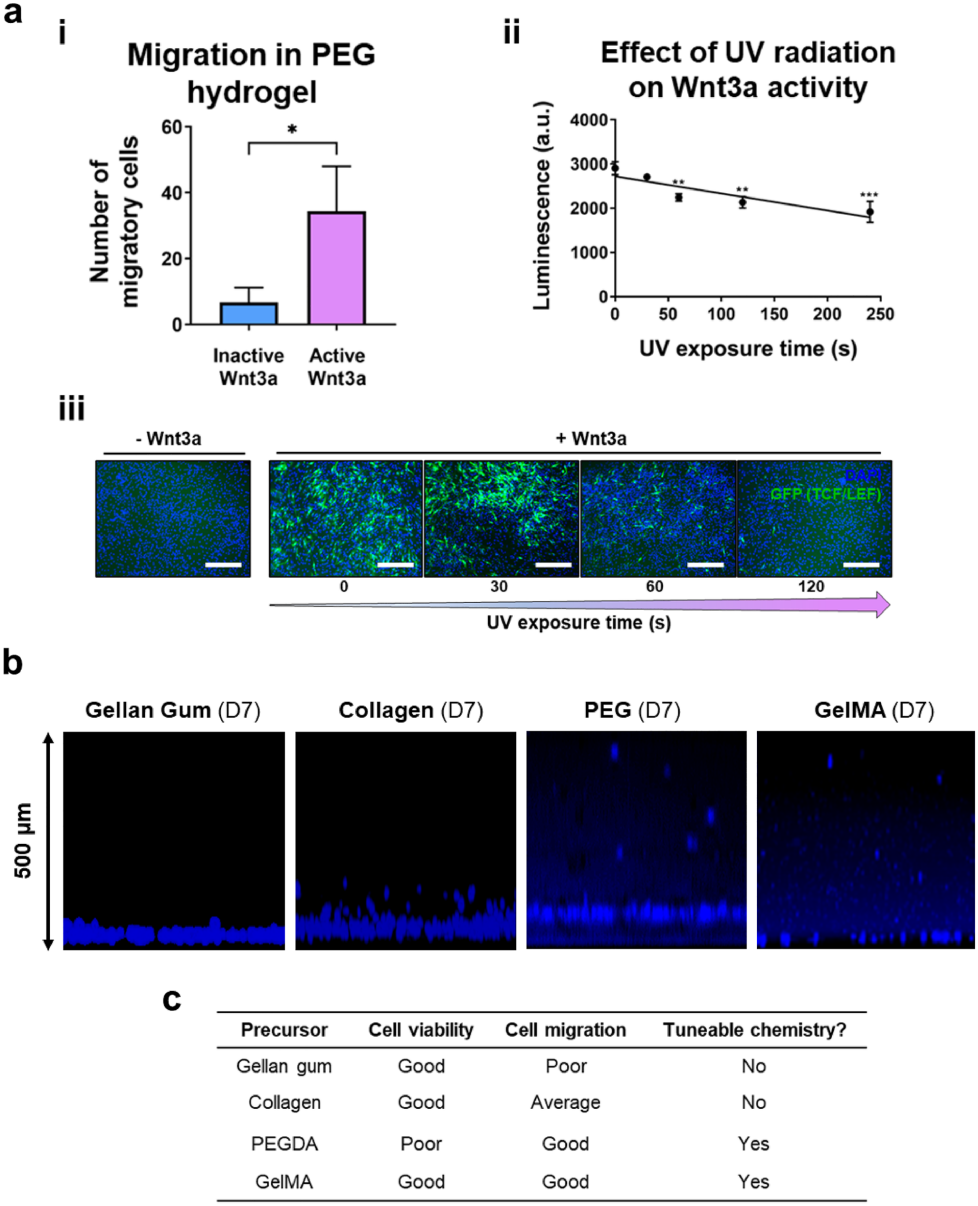
Screening of various hydrogels demonstrates that GelMA is optimal for the 3D regenerative cartilage model for drug screening applications. (**a**) Y201 cells in PEG hydrogel. (i) migration quantification, indicating that Y201s are able to migrate in PEG hydrogel (*p < 0.05). (ii-iii) UV radiation exposure during PEG curing negatively affects Wnt3a activity, as demonstrated by (ii) the decreasing luminescence (significance relevant to control (0 s); **p < 0.01; ***p < 0.001) and (iv) lower activation of GFP TCF/LEF reporter over increasing UV exposure (cell nuclei = blue; GFP TCF/LEF = green). Scale bars = 300 µm. (**b**) Representative immunofluorescence image of Y201 cells in different gels including gellan gum, collagen, PEG and GelMA hydrogel after 7 days of culture (nuclei = DAPI(blue)), showing poor cell migration in gellan gum towards the top of the gel, image of Y201 cells in collagen showing some level of cell migration towards the top of the gel, image of Y201 cells in PEG and gelMA hydrogel after 7 days of culture, showing good cell migration towards the top of the gel. (**c**) Table summarizing the properties of the four hydrogel systems screened, showing that gelMA outperforms all the other gels, due to its tuneable chemistry and capacity to sustain cell viability and migration.

Screening of different hydrogels was performed to identify the most appropriate system to sustain cell viability, migration, proliferation, and differentiation in the proposed 3D model (Fig. 1). When a PEG hydrogel is placed above the Y201 cell/Wnt monolayer, cells migrate (Fig. 3b) at a significantly higher rate in the presence of active Wnt3a compared to inactive Wnt3a (Fig. 3ai). However, exposure to UV radiation for gel curing was found to be detrimental to Wnt3a activity and cell viability, as shown in Fig. 3aii,iii. Gellan gum and collagen gels also demonstrated poor suitability for use in the 3D model. Indeed, both hydrogels present limited capability of chemistry tuneability, as summarized in Fig. 3c. Moreover, gellan gum limits cells’ migration (Fig. 3b) and collagen gels tend to contract during culture (Figure S1), although good cell migration and differentiation to the top of the system is observed. On the contrary, GelMA hydrogel presents the optimal properties amongst the screened systems, as summarized in Fig. 3c.

### Mechanical properties of GelMA hydrogel

**Figure 4:**
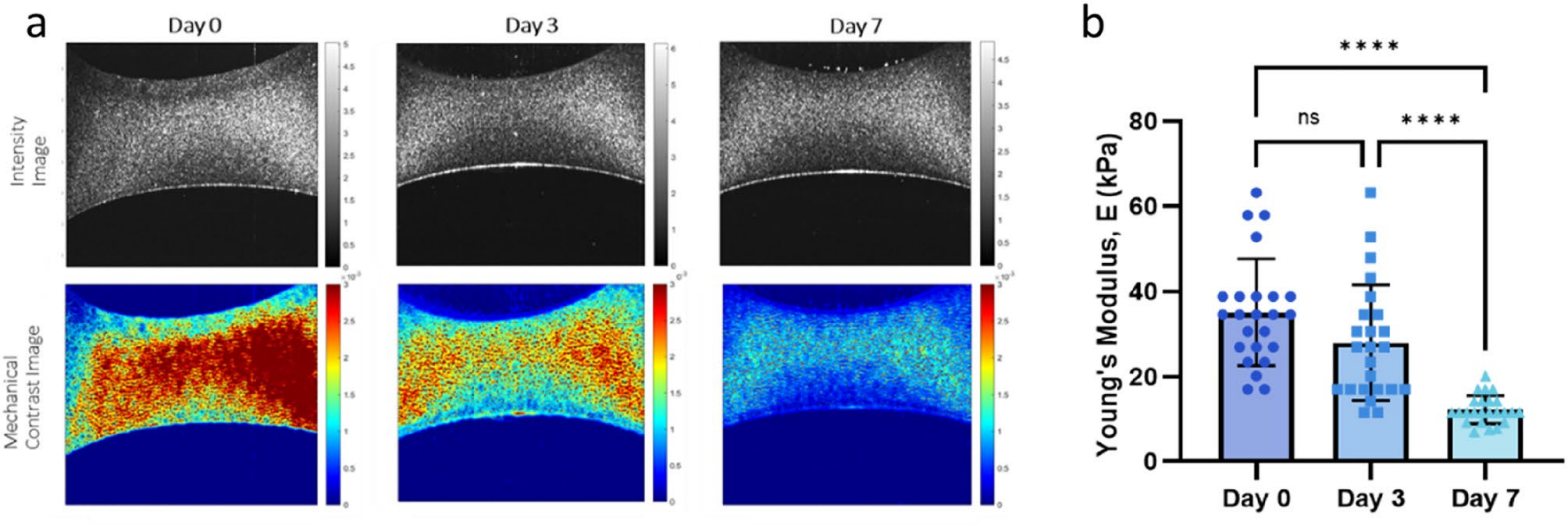
Mechanical profiling of the GelMA hydrogels following culture for 0,3 and 7 days. (**a**) Representations of depth imaging using intensity (top) and mechanical contrast (bottom) images of hydrogels on days 0, 3 and 7. (**b**) Calculated Young’s modulus for the homogenous 4% GelMA gels from debiased ambient vibration OCE analysis (^ns^*p* > 0.05; ****p < 0.0001).

We conducted debiased ambient vibration OCE analysis on 4% (w/v) GelMA hydrogels (n = 5) utilizing OCT testing on days 0, 3, and 7, as described in Section "Optical coherence tomography (OCT)". Notably, the observed decline in stiffness is prominently reflected in the mechanical contrast (λ) maps, as showcased in Fig. 4a. Furthermore, a comprehensive quantitative analysis of this stiffness reduction is shown in Fig. 4b, which presents the change in stiffness in Young's modulus. These figures offer a detailed insight into the mechanical properties of the tested hydrogels during the specified time points.

### Cell proliferation, migration and differentiation

**Figure 5:**
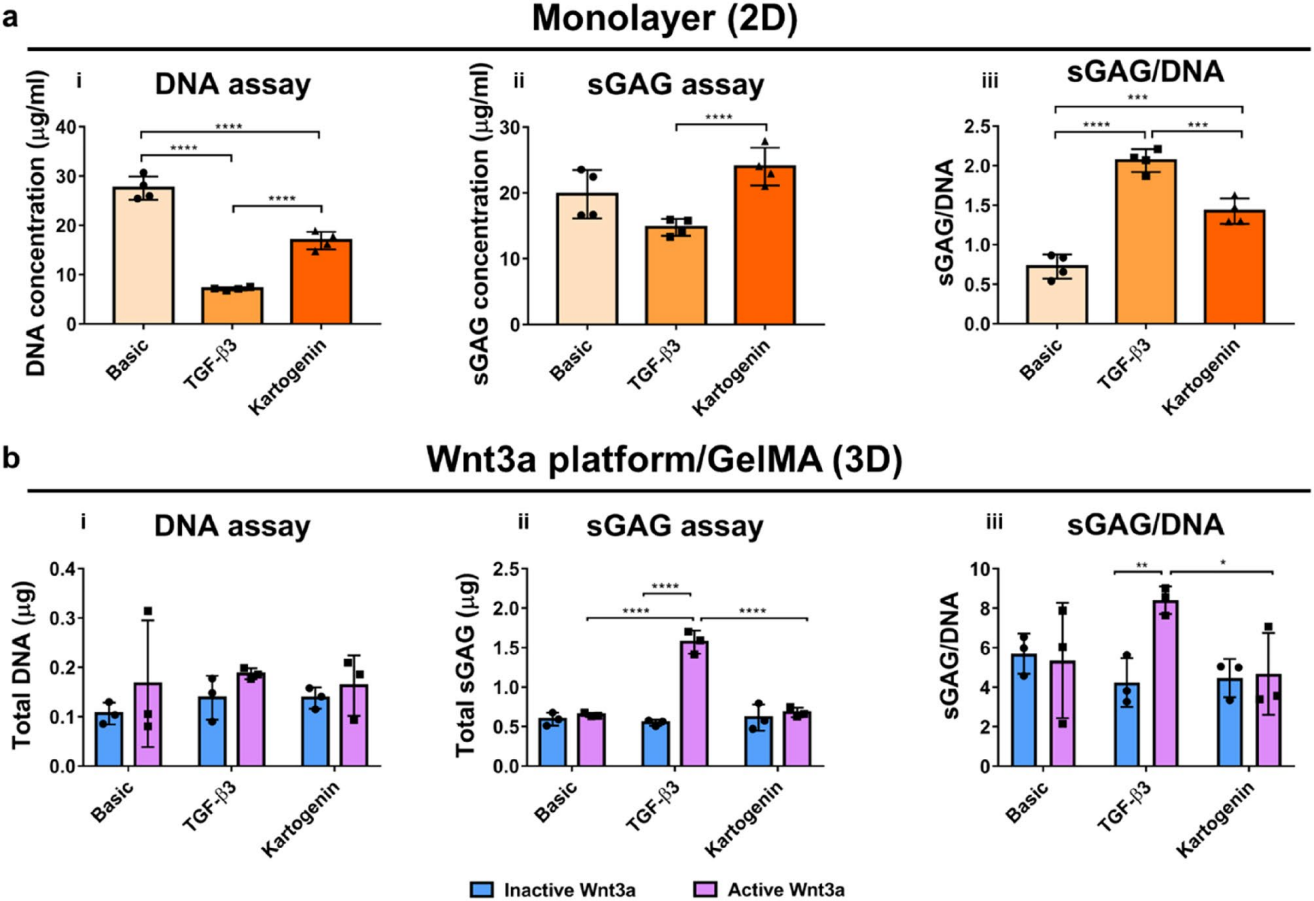
TGF-β3 and kartogenin have different chondro-inducive effects in 2D and 3D. (**a**) (i) DNA concentration, (ii) sGAG concentration and (iii) sGAG/DNA performed on Y201 cells cultured in monolayer (2D) indicate the potential chondro-inducive behavior of kartogenin in a 2D model (***p < 0.001; ****p < 0.0001). (**b**) (i) DNA content, (ii) sGAG production and (iii) sGAG/DNA performed on Y201 cells cultured in the Wnt3a platform/GelMA system indicate that TGF-β3 is chondro-inducive, while kartogenin does not enhance differentiation compared to basic condition in the 3D model (*p < 0.05; **p < 0.01; ****p < 0.0001).

Cell proliferation, migration and differentiation were analyzed to investigate the synergistic effect of immobilized Wnt3a platform and the chondro-inducive factors TGF-β3 and kartogenin. Y201 cells seeded in monolayer were also analyzed as positive controls into the effectiveness of the chondro-inducive compounds. Overall, both TGF-β3 and kartogenin significantly reduce cell proliferation compared to basic control (Fig. 5ai) when cells are cultured in monolayer, while no statistically significant differences are observed in the 3D model among all groups (Fig. 5bi). In particular, TGF-β3 has a remarkable effect in monolayer, inducing the lowest DNA concentration. A key marker for chondrogenic differentiation is sGAG production. In 2D models, kartogenin shows the highest amount of sGAG (Fig. 5aii), whereas this enhanced production is not observed in our 3D model (Fig. 5bii). In contrast with the addition of immobilized active Wnt3a, TGF-β3 both in monolayer (Fig. 5aiii) and in 3D (Fig. 5bii,iii) leads to a significant increase of total sGAG production and GAG production/cell. Taken together, these results show that a combination of TGF-β3 and immobilized active Wnt3a in GelMA hydrogel induces the highest amount of sGAG production. This data also demonstrates the different responses observed to these known chondrogenic agonists between 2 and 3D models.

**Figure 6:**
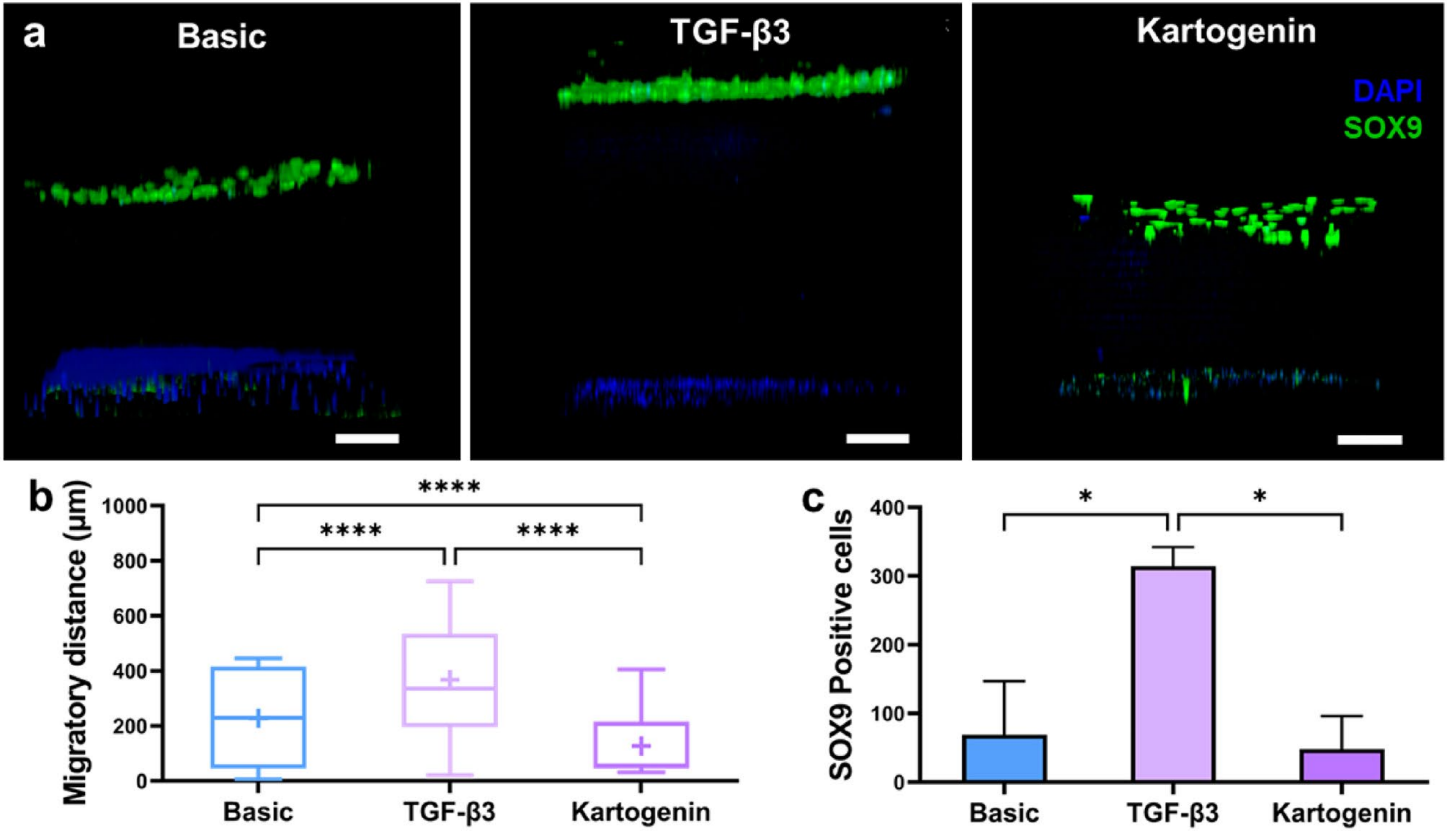
Cells migrate to the top of the gel and differentiate in the 3D regenerative model. (**a**) Representative immunofluorescence 3D image of Y201 cells after 7 days of culture, showing cell migration and differentiation towards the top of the gel in basic conditions (left), and in presence of TGF-β3 (center) and kartogenin (right) (nuclei = blue; SOX9 = green). Scale bars = 100 µm. (**b**) Quantification of cell migration, showing a significant increase in migratory distance in TGF-β3-treated samples compared to basic and kartogenin counterparts (data shown as median in interquartile range ± min/max and + symbol indicates mean value; ****p < 0.0001). (**c**) Quantification of SOX9 positive cells in the upper region of the gel shows increased differentiation in TGF-β3-treated samples compared to basic and kartogenin counterparts (*p < 0.05).

Generating complex 3D models enables the investigation of multiple properties of the chondrogenic differentiation and cartilage repair components. Alongside proliferation and sGAG production, the number of SOX9 positive and migrated Y201 cells was also assessed. As shown by confocal microscopy (Fig. 6a), the presence of immobilized Wnt3a promotes cell migration towards the top surface of the hydrogel. Y201 cells, when exposed to TGF-β3, migrate the furthest distance towards the surface of the gel (Fig. 6b). Interestingly, kartogenin seems to inhibit cell migration, as shown by a significant reduction in migratory distance compared to the basic control (Fig. 6b). Across all conditions, asymmetric division of cells can be observed, as SOX9 positivity is detected mainly in migratory cells (Fig. 6a). In accordance with sGAG production, addition of TGF-β3 significantly increases the number of SOX-9 positive cells compared to the basic control and addition of kartogenin, indicating that TGF-β3 has an inherently higher chondro-inducive effect than kartogenin (Fig. 6c).

## Discussion

Current 2D models for use in screening of drugs for diseases such as osteoarthritis have not been successful in identifying new potential therapeutics. The development of 3D regenerative cartilage models using hydrogels has gained significant attention in cartilage tissue engineering for its ability to better mimic the native cartilage microenvironment. The development of models that can enable the screening of a complex environment within cartilage would potentially enable new approaches to therapeutics to be defined. In this study, we successfully developed a 3D GelMA regenerative cartilage model incorporating bioactive cues, such as Wnt3a, and seeded with the bone marrow Y201 cell line. The model aimed to investigate the multiple components of chondrogenic repair including proliferation, differentiation, migration and cartilage-specific ECM production.

Our characterization of Y201 cells revealed their suitability as a scaleable and consistent cell component for the 3D cartilage model as demonstrated previously^26^. Under chondrogenic conditions, Y201 cells produced a cartilaginous matrix with increased proteoglycan content, and gene expression of chondrogenic markers ACAN and COL2A1 was upregulated, further confirming their chondrogenic potential^27^. Additionally, exposure to Wnt3a led to enhanced cell proliferation, indicating the activity of the Wnt/β-catenin pathway, which plays a critical role in cartilage development and regeneration, aligning with findings from other studies^13^. The activation of the TCF/LEF reporter by Wnt3a further supports the functionality of the Wnt signaling pathway in Y201 cells, similar to what has been reported^20^. Amongst the potential hydrogels screened, GelMA demonstrated the most appropriate properties, including tunable chemistry and support for cell viability and migration, making it the optimal choice for the 3D cartilage model. This finding is consistent with previous reports where GelMA hydrogels were shown to provide a suitable microenvironment for chondrogenic differentiation and support cartilage-specific ECM production^9,10^.

In the 3D cartilage model, TGF-β3 and kartogenin were used for validation and to investigate differences in responses between the 2D and 3D systems. While both compounds reduced cell proliferation in 2D monolayer culture, the 3D model showed no significant differences among all groups, indicating that the GelMA hydrogel platform supports cell viability and proliferation. These observations align with previous studies demonstrating the importance of the 3D environment in maintaining cell viability, migration and proliferation rates^5^. The initial mechanical properties of photo crosslinked GelMA closely resembled those reported in previous studies^7,34^. Notably, there was no significant reduction in the Young's modulus observed at day 3, indicating the hydrogels' stability during this critical initial period. This stability is crucial as it supports cell migration and the three-dimensional culture of cells. However, it's noteworthy that a significant reduction in mechanical properties became evident after 7 days. This decline can be attributed to the biodegradation of GelMA, a process governed by fundamental mechanisms, such as hydrolysis and enzymatic degradation^35,36^. In one of the studies, GelMA demonstrated a degradation rate when exposed to DPBS with a recorded weight loss of 19.56% after a 7-day period in the degradation assay^37^. In addition, Zhao et al.^38^ conducted a study to assess the degradation kinetics, in which GelMA hydrogels were immersed in a collagenase solution. The results of this investigation indicated a decrease in degradation rate with increasing GelMA concentrations. Specifically, it was observed that GelMA hydrogels containing as little as 5% GelMA achieved complete degradation in less than 3 days, while those with higher concentrations, such as 20% GelMA, displayed extended degradation periods of up to 8 weeks^38^. Consequently, given our choice of 7 days for evaluation, we observed a significant reduction in the mechanical properties of our GelMA models, aligning with the expected biodegradation trend observed in other studies. This observed decrease in mechanical properties over time highlights the biocompatible nature of GelMA for our cartilage tissue engineering models, as it allows the hydrogel to gradually yield to the produced ECM's mechanical properties to mimic native tissue properties.

Furthermore, TGF-β3 significantly increased the production of cartilage-specific ECM (sGAG) and the number of SOX9-positive chondrocytes, suggesting its chondrogenic potential within the 3D model. These results are consistent with other studies that have shown the chondro-inducive effects of TGF-β3 in promoting sGAG production and chondrogenic differentiation^25^. The effects of TGF-β3 were observed consistently across 2D and 3D models. Kartogenin, on the other hand, inhibited cell migration and showed limited enhancement of chondrogenic differentiation in the 3D models compared to the 2D, which is in line with studies reporting variable outcomes with kartogenin treatment^39,40^. The combination of TGF-β3 and immobilized active Wnt3a within the GelMA hydrogel showed enhanced chondrogenic differentiation, as evidenced by the increased sGAG production and SOX9-positive cell population. In our 3D model, we observed Y201 cell migration towards the upper surface of the gel system, accompanied by asymmetric cell division, providing evidence for chondrogenic commitment. These observations align with known Wnt3a effects reported in the literature, where it has been demonstrated to exert a significant influence on asymmetric cell division^18,19^ and cell migration^16,17^. In addition, these findings are also consistent with the potential synergistic effects of Wnt3a^41,42^ and TGF-β3 in promoting chondrogenic differentiation and ECM production^43,44^. Therefore, our findings align with previous reports on the suitability of GelMA hydrogel for cartilage tissue engineering, the chondrogenic potential of Y201 cells, and the role of Wnt3a and TGF-β3 in promoting chondrogenic differentiation and ECM production. Moreover, the comparison of our data with other studies highlights the unique advantages of our model, such as the integration of 3D architecture, the use of GelMA hydrogel, and the combination of Wnt3a and TGF-β3 for enhanced chondrogenic outcomes.

In conclusion, the 3D regenerative cartilage model developed in this study offers a valuable tool for investigating cartilage biology, disease mechanisms, and for scale-up in drug screening applications. Our data support the potential of this model for cartilage tissue engineering and provide insights into cartilage regeneration mechanisms in a cartilage repair environment. Furthermore, we have validated the robustness of our model and its potential to discover novel therapeutic approaches for cartilage repair and osteoarthritis treatment. Continued optimization and validation of this model will contribute to the advancement of cartilage tissue engineering and accelerate the development of effective therapies for cartilage regeneration in response to diseases such as Osteoarthritis.

### Supplementary Information

Supplementary Figures.

## Data Availability

The datasets used and/or analysed during the current study available from the corresponding author on reasonable request.
