# Supplementary material for: Enhanced chondrogenic potential in GelMA-based 3D cartilage model via Wnt3a surface immobilization
Source: Sci Rep. 2024 Jul 1;14:15022. doi: 10.1038/s41598-024-65970-w (PMC11217376; doi:10.1038/s41598-024-65970-w)
Supplement: Supplementary file 1 — Supplementary Figures. [file 41598_2024_65970_MOESM1_ESM.docx]

**Supporting Information**

**Enhanced Chondrogenic Potential in GelMA-Based 3D Cartilage Model via Wnt3a Surface Immobilization**

Angela Imere^a†^, Nicola C. Foster^a†^, Hadi Hajiali^a†*^, Kerime Ebrar Okur^a^, Abigail L. Wright^a^, Ines A. Barroso^a^ and Alicia J. El Haj^a*^

*^a^Healthcare Technologies Institute,* *Institute of Translational Medicine, National Institute for Health and Care Research (NIHR) Birmingham Biomedical Research Centre, School of Chemical Engineering, University of Birmingham, Birmingham, B15 2TT, UK*

^†^ These authors are Joint first authors.

*Corresponding authors: [h.hajiali@bham.ac.uk](mailto:h.hajiali@bham.ac.uk); [a.elhaj@bham.ac.uk](mailto:a.elhaj@bham.ac.uk)

**
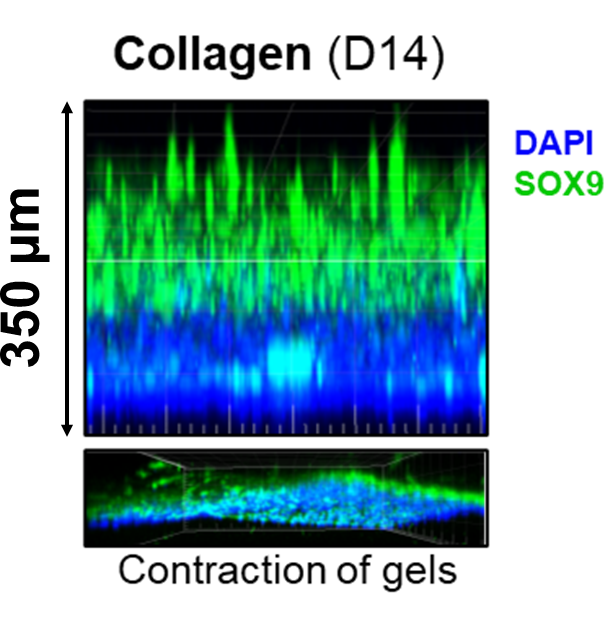
**

**Figure S1:** Representative immunofluorescence image of Y201 cells in collagen hydrogel after 14 days of culture showing cell migration and differentiation towards the top of the gel but poor stability and strong contraction of the gel (nuclei = blue; SOX9 = green).
